# Utilization of Face-to-Face Vestibular Support Groups: A Comparison to Online Group Participation

**DOI:** 10.1177/00034894241241861

**Published:** 2024-05-13

**Authors:** Erik B. Vanstrum, Min Jung Kim, Ryan S. Ziltzer, Joni K. Doherty, Alaina M. Bassett

**Affiliations:** 1 Department of Head and Neck Surgery, David Geffen School of Medicine at the University of California Los Angeles, Los Angeles, CA, USA; 2 David Geffen School of Medicine at the University of California Los Angeles, Los Angeles, CA, USA; 3 Keck School of Medicine, Los Angeles, CA, USA; 4 Caruso Department of Otolaryngology—Head and Neck Surgery, University of Southern California, Los Angeles, CA, USA; 5 California State University, Los Angeles, CA, USA

**Keywords:** vestibular disorders, support groups, online support groups, face-to-face support groups, coping strategies, social support

## Abstract

**Objective:**

This study compared the utilization and outcomes of face-to-face (F2F) vestibular support groups and online support communities (OSC) for individuals with vestibular disorders.

**Methods:**

We distributed a 31-question anonymous electronic survey through the Vestibular Disorders Association (VeDA) to F2F participants, categorizing user involvement in F2F, OSCs, or both and assessed impact on medical decision-making, psychosocial benefits, and goals achieved.

**Results:**

The F2F cohort consisted of 97 individuals comprising primarily of non-Hispanic White women (mean age = 57 years, SD ± 14 years) with diagnoses including persistent postural-perceptual dizziness (19%), Meniere’s disease (15%), and vestibular neuritis (13%). Most participants were diagnosed by an otolaryngologist (65%) and attended F2F meetings monthly or less frequently (78%). The OSC group comprised of 551 individuals, primarily of non-Hispanic White women, but was younger in age (mean age = 50 years, SD ± 13 years). OSC participants notably engaged more, with 36% participating on a daily basis and 32% multiple times a week. F2F participants were older (mean age 57 years vs 50 years, *P* < .001) and more commonly referred by medical professionals (22% F2F vs 6% OSC, *P* < .001). Both groups had similar achieved goals, including hearing from others with the same diagnosis (84% vs 89%, *P* > .05) and similar impact on medical decision-making (75% vs 78%, *P* > .05). More F2F participants reported increased development of coping skills (79% F2F vs 69% OSC, *P* = .037). OSC participants typically found the group via an online search (75%), compared to 51% for F2F. OSC participants had higher daily engagement (36%) compared to F2F (1%).

**Conclusion:**

F2F users are older and more commonly referred by medical professionals. Despite less frequent engagement, F2F participants reported similar influences on achieved goals, medical decision-making, and impact on psychosocial benefits. These findings highlight the importance of both F2F and OSC support groups for individuals with vestibular disorders.

## Introduction

Vestibular illness is common in the U.S., with objective measures of imbalance suggesting that as many as 35% of adults over 40 have a vestibular deficit.^1^ However, the medical community still faces challenges in diagnosing and managing these patients. Despite annual medical expenditure exceeding $48 billion for patients experiencing vertigo and dizziness,^2^ only 1 in 5 patients with vestibular illness believe they received a timely and accurate diagnosis.^3^ Navigating uncertain medical terrain while sustaining high healthcare costs is compounded at the individual level by challenges intrinsic to living with vestibular dysfunction. Intrusive symptoms, such as dizziness and imbalance, may arise unexpectedly and significantly impact activities of daily living. Furthermore, the association between vestibular disorders and mental health comorbidities is widely documented.^4-8^

Given these challenges, it is unsurprising that patients experiencing vestibular dysfunction have sought alternative resources. Vestibular support groups are organized online and in person, often by patients with vestibular diagnoses, and also by associated health care providers such as physical therapists. These groups can target general audiences, for example, *Vestibular Disorders Support Group*, or specific patient populations, for example, *Meniere’s Disease Support Group*. In person support groups have an international presence, including throughout the U.S., Canada, Australia, and the UK.

Our group recently reported on the widespread use of online support communities (OSC) among patients with vestibular dysfunction.^9^ We showed that hundreds of thousands of individuals engage with OSCs across vestibular diagnoses. A surveyed cohort of over 500 users suggests that most OSC participants have engaged with their groups daily for multiple years. Furthermore, 9 in 10 participants cited peer support—sharing the illness experience—as a primary reason for joining. This work is consistent with previous studies which suggest that support groups offer important resources such as peer support, offering coping mechanisms, sharing educational resources for those with challenging medical diagnoses, such as cancer.^10,11^

Similarly, face-to-face (F2F) support groups play a significant role for many individuals with vestibular disorders. However, compared to OSCs, these groups tend to have a different demographic, with older participants who meet less frequently. F2F participants often benefit from direct social interaction, which could have distinct advantages in terms of emotional support and shared experiences.

The use and influence of vestibular F2F support groups remains unclear; there are only a few reports evaluating differences between users of F2F groups and OSCs, and only in the oncologic setting.^12,13^ The goals of this project are to explore clinicodemographic features of F2F vestibular group users, evaluate the influence of F2F groups on psychosocial outcomes and medical decision-making, and compare these metrics to users of vestibular OSCs.

## Materials and Methods

This study qualified for exemption by the institutional review board at the University of Southern California (UP-21-00483).

### Survey Development

The survey was developed in conjunction with published questionnaires previously used to query characteristics and user perceptions of support groups unrelated to vestibular illness.^10,14^ These survey instruments were not tested for reliability or validity. A team comprised of patients, vestibular support group leaders, otolaryngologists, and a vestibular audiologist provided feedback to develop survey content and structure. The resulting survey included 31 questions and was designed to be completed in less than 10 minutes. The survey was not tested for reliability or validity. It used branching logic to ask questions specific to the participant’s involvement in F2F, OSCs, or both styles of support groups.

### Survey Distribution

Support group leaders were identified through their association with the non-profit organization Vestibular Disorders Association (VeDA; vestibular.org), which offers a forum for users to connect with local support groups, among other resources. An invitation to participate was sent to 23 group leaders; 9 group leaders responded affirmatively, agreeing to share the survey among their active members. The exact number of members to whom the survey was distributed to is unknown. The survey was shared electronically using Qualtrics (Qualtrics, Provo, UT), and participants were not offered compensation.

A manuscript previously published by the authors reports in detail on the methods and results for OSC participation, and the survey questions can be found as a supplement to this article.^9^ Briefly, the survey was distributed to 2 OSCs following approval from group moderators. Each OSC targeted patients with general vestibular symptoms as opposed to those that target specific diagnoses. These 2 groups included over 20 000 members.

### Statistical Analysis

Only complete surveys were included for final analysis. Descriptive statistics were calculated in Excel (version 16.53). Important outcomes were assessed on a Likert scale and related to achieved goals after group participation, impact on medical decision-making, and perceived influence of psychosocial benefit. These variables were dichotomized (≤3 vs ≥4) to enhance analytic power. Independent *t*-test and Fisher’s exact method were used to compare differences between F2F and OSC group participants. These calculations were performed using SPSS 27 (IBM Corp., Chicago) with significance defined as *P* < .05.

## Results

### Clinicodemographic Profile of F2F Participants

The majority of the sample were non-Hispanic White individuals (93%; Table 1) with a mean age of 57 years (SD ± 14 years). Most participants held a college degree (35%) or a postgraduate degree (40%). The sample was well-represented globally (40% of respondents were outside of the US) and geographically dispersed throughout the US, with the highest representation within the US coming from the West and Midwest Regions (21% each).

**Table 1.** Demographic Information of F2F Users.

No. of participants	97
Mean age (years ± SD)	57 ± 14
Gender n (%)	
Female	76 (78)
Male	20 (21)
Non-binary	1 (1)
Race/ethnicity n (%)	
Asian/Pacific Islander	2 (2)
Non-Hispanic White	90 (93)
Other	5 (5)
Geographic region n (%)	
East Coast	12 (12)
Midwest	20 (21)
West Coast	20 (21)
South	6 (6)
International	39 (40)
Education n (%)	
High school diploma or GED	4 (4)
Vocational training	3 (3)
Some college	17 (18)
College degree	34 (35)
Graduate degree	39 (40)

Self-reported clinical information is presented in Table 2. The most common self-reported diagnoses among participants included persistent postural-perceptual dizziness (19%), Meniere’s disease (15%), vestibular neuritis (13%), and vestibular migraine (12%). The majority of participants reported having a secondary vestibular diagnosis (59%); most had received their primary diagnosis more than 5 years prior (55%) or between 1 and 5 years prior (37%). An otolaryngologist had diagnosed most of the participants (65%) with vestibular disorders.

**Table 2.** Clinical Information of F2F Users.

No. of participants	97
Primary diagnoses* n (%)	
Persistent postural perceptual dizziness	18 (19)
Meniere’s disease	15 (15)
Vestibular neuritis	13 (13)
Vestibular migraine	12 (12)
Other	10 (10)
Bilateral vestibular hypofunction	6 (6)
Undiagnosed	5 (5)
Benign paroxysmal positional vertigo	4 (4)
Labyrinthitis	3 (3)
Mal de debarquement	3 (3)
Secondary diagnoses^#^ n (%)	
Vestibular migraine	17 (18)
Benign paroxysmal positional vertigo	16 (16)
Other	15 (15)
Vestibular neuritis	10 (10)
Persistent postural perceptual dizziness	8 (8)
Time since diagnosis n (%)	
<3 months	0 (0)
3 months to 1 year	7 (7)
>1 to 5 years	36 (37)
>5 years	53 (55)
Medical providers seen prior to diagnosis n (%)	
1-4	59 (61)
5-9	29 (30)
10+	9 (9)
Diagnosing specialist n (%)	
Audiologist/vestibular specialist	2 (2)
Emergency medicine physician	1 (1)
Neurologist	19 (20)
No formal diagnosis	2 (2)
Other	2 (2)
Otolaryngologist	63 (65)
Physical therapist	6 (6)
Primary care provider	2 (2)
Treatment n (%)	
Dietary changes	41 (42)
Epley maneuver	35 (36)
Medication	60 (62)
Other	9 (9)
Surgery	14 (14)
Vestibular rehabilitation or physical therapy	86 (89)

Top 10* and top 5^#^ diagnoses.

### F2F Support Group Participation

Measures of F2F support group participation are shown in Table 3. More than half of the participants reported a length of membership between 1 and 5 years (51%). Engagement with the support groups occurred on a monthly basis (30%) or once every few months (48%). The most frequent informational sources for group discovery were online search (51%) and a medical-professional recommendation (22%).

**Table 3.** F2F Participation.

No. of participants	97
Length of membership n (%)	
<3 months	14 (14)
3 months to 1 year	22 (23)
>1 year to 5 years	49 (51)
> 5 years	12 (12)
Level of engagement n (%)	
Daily	1 (1)
Multiple times per week	2 (2)
Weekly	11 (11)
Monthly	29 (30)
Once every few months	47 (48)
Other	7 (7)
Information source about group n (%)	
Online search	49 (51)
Recommended by medical professional	21 (22)
Word of mouth	15 (15)
Other	15 (15)

Most respondents believed that F2F support groups protected their privacy (84%), trusted the information provided within each group (85%), and provided a safe place to share personal experiences (91%). Similarly, most participants indicated that they would recommend joining a support group to others with their condition (90%).

### F2F and OSC Comparison: Clinicodemographic and Group Participation

Clinicodemographic features and measures of support group participation were compared between users of F2F groups and OSCs (Supplemental Table 1). OSC participants were significantly younger than F2F participants (mean age = 50 years vs 57 years, *P* < .001) and more commonly female (89% OSC vs 78% F2F, *P* = .039). There were no differences in time to receiving a primary diagnosis and number of providers seen prior to receiving a diagnosis. F2F groups were attended less frequently by users and were more commonly recommended by medical professionals (22% F2F vs 6% OSC; χ^2^ = 0.20, df *=* 3, *P* < .001)

### F2F and OSC Comparison: Vestibular Support Group Outcomes

**Figure 1.**
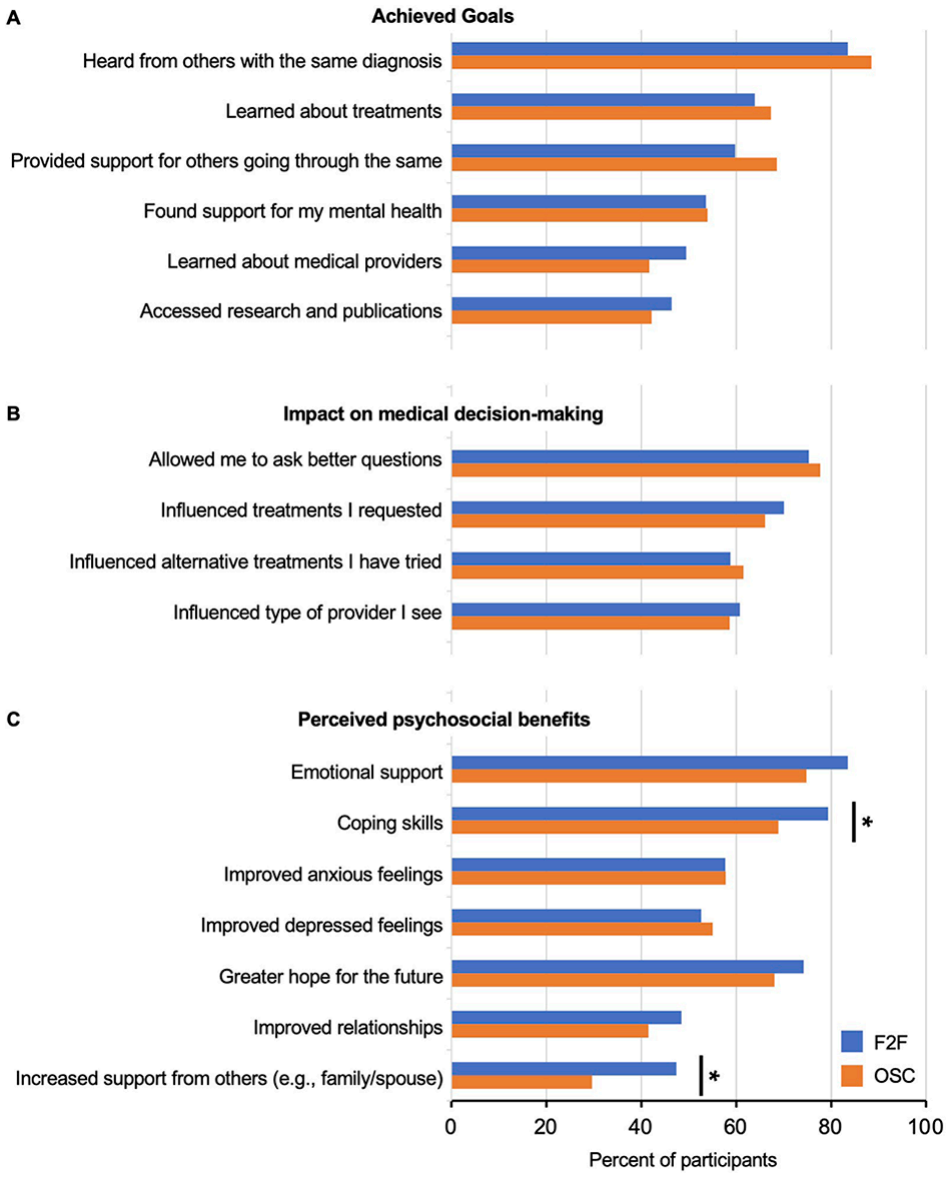
Comparison of support group outcomes for F2F group users (n = 97) and OSC users (n = 602). (A) Achieved goals. (B) Impact on medica decision-making. (C) Perceived psychosocial benefits.

Similar proportions of respondents reported achieving their goals in vestibular support group participation whether they were attending F2F groups or OSCs (Figure 1A). Achieved goals included hearing from others with the same diagnosis (84% vs 89%, χ^2^ = 1.98, df = 1, *P* > .05; F2F vs OSC), learning about treatment options (64% vs 67% χ^2^ = 0.20, df *=* 1, *P* > .05), and accessing research publications (46% vs 42%, χ^2^ = 0.60, df = 1, *P* > .05). Additionally, F2F users reported similar impact on medical decision-making compared with OSC users (Figure 1B). F2F participants believed that involvement in their group allowed them to ask better questions to medical providers (75% vs 78%, χ^2^ = 0.29, df = 1, *P* > .05; F2F vs OSC, respectively) and make treatment requests (70% vs 66%, χ^2^ = 0.59, df = 1, *P* > .05).

While F2F and OSC users cited similar perceived psychosocial benefits, a higher proportion of F2F participants reported increased coping skills and that their spouse and/or family developed new ideas for their support after group participation (79% vs 69%, χ^2^ = 4.37, df = 1, *P* = .037; 47% vs 30%, χ^2^ = 12.2, df = 1, *P* < .001; F2F vs OSC) (Figure 1C). A higher proportion of F2F users also cited increased emotional support, although this relationship was not significant (84% vs 75%, χ^2^ = 3.51, df = 1, *P* = .072).

### Users of Both F2F and OSCs

A sub-analysis of the 53 individuals that reported using both F2F and OSC groups was performed (Supplemental Figure 1). These participants completed support group outcomes data for each type of group. There were no significant differences in the outcomes queried, with the exception that a higher proportion of participants reported that OSC groups allowed for increased ability to provide support for others with vestibular illness (87% vs 66%, *P* = .003).

## Discussion

Patients with vestibular illness face both extrinsic hurdles (eg, significant healthcare costs and difficulty in receiving a diagnosis) and intrinsic hurdles (eg, impact of dizziness on activities of daily living and illness uncertainty). Considering such difficulties, these patients may seek additional help in the form of peer-support. This study reports on the clinicodemographic features of F2F support group members for patients with vestibular diagnoses and highlights the perceived influence of peer support groups on psychosocial factors and medical decision-making. Vestibular F2F group participants most often participate in meetings monthly or bimonthly and demonstrate multi-year long commitment. The diverse clinical profile of the F2F cohort suggests that vestibular symptoms, rather than diagnoses, are the unifying patient feature upon which peer-support is centered. Comparing perceived benefits of F2F group participation to online vestibular community users, there is a similar influence on achieved goals and impact on medical decision-making. However, F2F participation compared with OSC use may offer an increased development of coping skills and support from others such as family or a spouse.

Numerous support group studies, involving both online and F2F groups, contain populations which are predominately female.^15-18^ In a cohort of 505 support group participants seeking medical care for diverse etiologies, 85% were female and 93.5% were non-Hispanic white. This demographic profile is consistent with this study and the vestibular OSC population.^9^ It is well-documented that female patients more commonly present with dizziness and consult with a physician for dizziness or vertigo.^19,20^ However, it is unclear why this racial population is overrepresented despite vestibular illness impacting a diversity of racial/ethnic backgrounds.^1^ Further research into why underrepresented populations may not be utilizing these resources is merited.

### Differences Between Vestibular F2F Groups and OSCs

Few studies have directly compared the use of F2F groups and OSCs. Our study shows that vestibular F2F users are, on average, older than OSC participants. This finding is consistent with previous studies that suggest older populations may be less technologically savvy.^12,13^ While Huber et al^12^ reported that OSC users were more educated than F2F users, our findings showed no differences in education level among vestibular support group users.

Both Huber et al.^12^ and Setoyama et al.^13^ evaluated anxiety and depression in F2F versus OSC users using validated measures such as PHQ-2, GAD-2, and HADS. Huber found no difference in anxiety or depression scores between the 2 groups, whereas Huber et al^12^ and Setoyama et al^13^ found a slight decrease in anxiety among OSC users but no difference in depression. Our study is consistent with these findings, showing no significant difference in improved anxious or depressed feelings among vestibular F2F versus OSC users. However, we found that a higher proportion of F2F users cited increased learned coping skills from participation, which is also consistent with previous studies.^13^

While F2F users are participating once per month or less frequently, OSC users participate daily. Despite this disparity in engagement frequency, our study found similar achieved goals, influence on medical decision-making, and psychosocial benefits reported by both F2F and OSC users. These results are further supported by the sub-analysis of F2F users who also participate in OSCs. Thus, from the patient’s perspective, participation in F2F groups may create similar benefits but with significantly reduced time expenditure. Our results suggest that F2F support groups may provide additional benefits, such as increased coping skills and support from loved ones/family members, which online participation lacks. F2F support groups may provide added benefit in creating avenues for participation among family or loved ones. For certain patient populations, such as the elderly, F2F groups may be more accessible. While others have argued that these 2 resources are complementary,^13^ our results suggest that vestibular patients seeking peer support may receive similar benefits from participation in either.

Nearly a quarter of F2F group users were recommended to their group by a medical professional, compared to only 6% of the OSC cohort. Despite concerns of sharing of misinformation, the medical validity of peer-support, and the potential for psychological harm, attitudes have shifted over time and there is growing familiarity with support groups and participation, especially among oncologic medical providers.^21-23^ In a study, the majority of prostate cancer specialists (urologists, radiation oncologists, and oncologists) viewed face-to-face groups positively with approximately two-thirds participating to some degree. However, this cohort raised concerns about the utility of online groups, including accessibility issues for older patients, miscommunication, and hostility of other members. Currently, no studies have evaluated the medical validity of information/advice-seeking shared on online forums.

This cross-sectional survey study has several limitations. Since the start of the COVID-19 pandemic, many F2F support groups have transitioned into the online space using platforms such as Zoom; we did not distinguish between these forms of F2F support. However, it may be hypothesized that there are differences in online versus in-person F2F interactions (eg, the number of interactions or length of interactions). Further, distribution by F2F group leaders did not allow for calculation of response rate or evaluation of non-response bias. Our study also did not use validated measures to calculate vestibular, quality of life, or mental health measurements. Further, this study relied upon a survey instrument that was not tested for validity or reliability. Our results are consistent with previous studies that have applied these tools.^12,13^ Lastly, this study was administered only in English-to-English speaking support groups. Despite these limitations, our results add to the small and investigatory body of literature on peer-support resources for patients with vestibular illness. Future studies of vestibular support groups should evaluate the longitudinal influence of peer-support using validated measures of dizziness (DHI) or mental health co-morbidities and whether peer-support can positively influence treatment outcomes such as vestibular rehabilitation.

## Conclusion

This study provides valuable insights on the clinicodemographic features and outcomes of F2F support group use among patients with vestibular disorders. Our findings show that, compared to OSC users, F2F users tend to be older and are more frequently recommended to participate by medical professionals. Despite engaging significantly less frequently with support groups (1% of F2F users engaging daily compared to 36% of OSC users), F2F users report similar influences on achieved goals and medical decision-making. F2F participants may derive additional benefits from an increased development of coping skills and receiving support from family or loved ones. Overall, our study suggests that F2F support groups can be a valuable resource for patients with vestibular illnesses, providing unique benefits that may not be available through online support communities. Both types of support groups are beneficial for individuals with vestibular disorders; F2F groups may be more suitable for those who require more emotional and social support, as 79% of F2F users reported increased development of coping skills compared to 69% of OSC users (*P* = .037), whereas OSCs may be more appropriate for younger individuals and those who require more practical and informational support, with 75% of OSC users having found the group through an online search compared to 51% of F2F users.

## Supplemental Material

sj-docx-1-aor-10.1177_00034894241241861 – Supplemental material for Utilization of Face-to-Face Vestibular Support Groups: A Comparison to Online Group Participation Supplemental material, sj-docx-1-aor-10.1177_00034894241241861 for Utilization of Face-to-Face Vestibular Support Groups: A Comparison to Online Group Participation by Erik B. Vanstrum, Min Jung Kim, Ryan S. Ziltzer, Joni K. Doherty and Alaina M. Bassett in Annals of Otology, Rhinology & Laryngology

sj-jpg-2-aor-10.1177_00034894241241861 – Supplemental material for Utilization of Face-to-Face Vestibular Support Groups: A Comparison to Online Group Participation Supplemental material, sj-jpg-2-aor-10.1177_00034894241241861 for Utilization of Face-to-Face Vestibular Support Groups: A Comparison to Online Group Participation by Erik B. Vanstrum, Min Jung Kim, Ryan S. Ziltzer, Joni K. Doherty and Alaina M. Bassett in Annals of Otology, Rhinology & Laryngology
