# Supplementary material for: Utilization of Face-to-Face Vestibular Support Groups: A Comparison to Online Group Participation
Source: Ann Otol Rhinol Laryngol. 2024 May 13;133(8):713–9. doi: 10.1177/00034894241241861 (PMC11290019; doi:10.1177/00034894241241861)
Supplement: sj-docx-1-aor-10.1177_00034894241241861 – Supplemental material for Utilization of Face-to-Face Vestibular Support Groups: A Comparison to Online Group Participation [file sj-docx-1-aor-10.1177_00034894241241861.docx]

| **Supplemental Table 1.** Comparison of F2F and OSC clinicodemographic data | | | | | | |
| --- | --- | --- | --- | --- | --- | --- |
|  | F2F (n = 97) | |  | OSC (n = 551) | |  |
|  | n | % |  | n | % | p-value |
| **Age**, mean ± SD | 57 ± 14 | - |  | 50 ± 13 | - | <0.001^#^ |
| **Gender** |  |  |  |  |  | 0.039^*^ |
| Female | 76 | 78% |  | 488 | 89% |  |
| Male | 20 | 21% |  | 59 | 11% |  |
| **Race/Ethnicity** |  |  |  |  |  | 0.011^*^ |
| **Geographic region** |  |  |  |  |  | <0.001^*^ |
| East Coast | 12 | 12% |  | 162 | 30% |  |
| Midwest | 20 | 21% |  | 108 | 20% |  |
| West Coast | 20 | 21% |  | 68 | 12% |  |
| South | 6 | 6% |  | 86 | 16% |  |
| International | 39 | 40% |  | 125 | 23% |  |
| **Education** |  |  |  |  |  | 0.289^*^ |
| High school diploma or GED | 4 | 4% |  | 58 | 11% |  |
| Vocational training | 3 | 3% |  | 22 | 4% |  |
| Some college | 17 | 18% |  | 104 | 19% |  |
| College degree | 34 | 35% |  | 194 | 35% |  |
| Graduate degree | 39 | 40% |  | 170 | 31% |  |
| **Primary Diagnoses** |  |  |  |  |  | <0.001^*^ |
| **Time Since Diagnosis** |  |  |  |  |  | 0.158^*^ |
| < 3 months | 0 | 0% |  | 9 | 2% |  |
| 3 months to 1 year | 7 | 7% |  | 51 | 9% |  |
| > 1 year to 5 years | 36 | 37% |  | 247 | 45% |  |
| > 5 years | 53 | 55% |  | 242 | 44% |  |
| **Medical Providers Seen Prior to Diagnosis** |  |  |  |  |  | 0.411^*^ |
| 1-4 | 59 | 61% |  | 315 | 57% |  |
| 5-9 | 29 | 30% |  | 178 | 32% |  |
| 10+ | 9 | 9% |  | 56 | 10% |  |
| **Diagnosing Specialist** |  |  |  |  |  | 0.03^*^ |
| Neurologist | 19 | 20% |  | 143 | 26% |  |
| Otolaryngologist | 63 | 65% |  | 278 | 51% |  |
| Other | 15 | 15% |  | 128 | 23% |  |
| **Length of membership** |  |  |  |  |  | 0.87^*^ |
| < 3 months | 14 | 14% |  | 64 | 12% |  |
| 3 months to 1 year | 22 | 23% |  | 120 | 22% |  |
| > 1 year to 5 years | 49 | 51% |  | 294 | 54% |  |
| > 5 years | 12 | 12% |  | 71 | 13% |  |
| **Level of engagement** |  |  |  |  |  | <0.001^*^ |
| Daily | 1 | 1% |  | 198 | 36% |  |
| Multiple times per week | 2 | 2% |  | 173 | 32% |  |
| Weekly | 11 | 11% |  | 84 | 15% |  |
| Monthly | 29 | 30% |  | 46 | 8% |  |
| Once every few months | 47 | 48% |  | 42 | 8% |  |
| Other | 7 | 7% |  | 6 | 1% |  |
| **Information source about group** |  |  |  |  |  | <0.001^*^ |
| Online search | 49 | 51% |  | 413 | 75% |  |
| Recommended by medical professional | 21 | 22% |  | 33 | 6% |  |
| Word of mouth | 15 | 15% |  | 42 | 8% |  |
| Other | 15 | 15% |  | 95 | 17% |  |
| ^#^t-test |  |  |  |  |  |  |
| ^*^Chi-square |  |  |  |  |  |  |
