# Supplementary figures and images for: Utilization of Face-to-Face Vestibular Support Groups: A Comparison to Online Group Participation
Source: Ann Otol Rhinol Laryngol. 2024 May 13;133(8):713–9. doi: 10.1177/00034894241241861 (PMC11290019; doi:10.1177/00034894241241861)

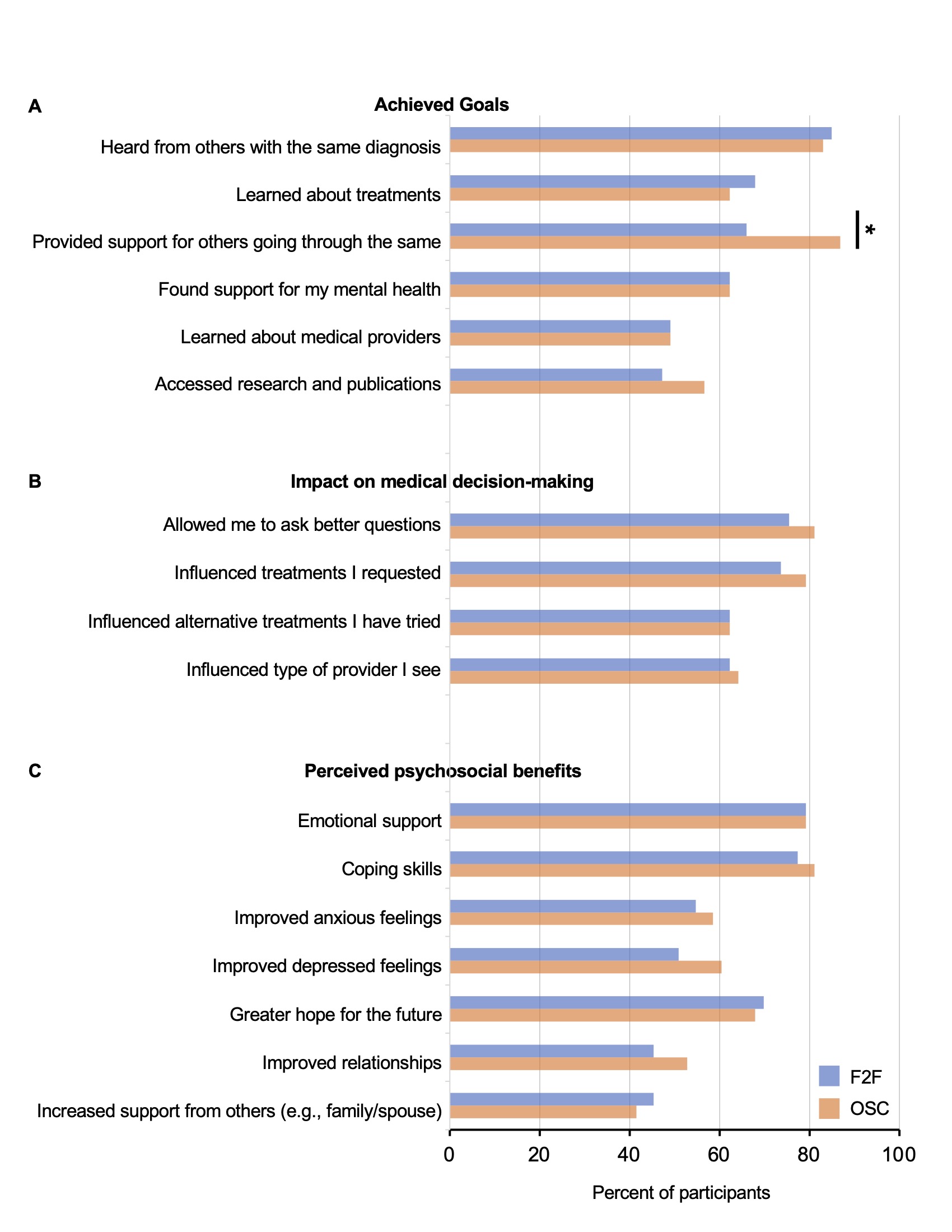

Supplement: sj-jpg-2-aor-10.1177_00034894241241861 – Supplemental material for Utilization of Face-to-Face Vestibular Support Groups: A Comparison to Online Group Participation [file sj-jpg-2-aor-10.1177_00034894241241861.jpg]
